# Sustainable Valorization of Mussel Shell Waste: Processing for Calcium Carbonate Recovery and Hydroxyapatite Production

**DOI:** 10.3390/jfb17010024

**Published:** 2025-12-30

**Authors:** Adriana Poli Castilho Dugaich, Andressa da Silva Barboza, Marianna Gimenes e Silva, Andressa Baptista Nörnberg, Marcelo Maraschin, Maurício Malheiros Badaró, Daiara Floriano da Silva, Carlos Eduardo Maduro de Campos, Carolina dos Santos Santinoni, Sheila Cristina Stolf, Rafael Guerra Lund, Juliana Silva Ribeiro de Andrade

**Affiliations:** 1 Department of Odontology, Federal University of Santa Catarina, UFSC, Florianópolis 88040-900, SC, Brazil; adrianapoli@gmail.com (A.P.C.D.); andressahb@hotmail.com (A.d.S.B.); gimenesmarianna@gmail.com (M.G.e.S.); mauricio.badaro@ufsc.br (M.M.B.); carolina.santinoni@ufsc.br (C.d.S.S.); sheila.stolf@ufsc.br (S.C.S.); 2 Laboratory of Technology and Development of Composites and Polymeric Materials (LaCoPol), Center of Chemical, Pharmaceutical and Food Sciences, Federal University of Pelotas (UFPel), Campus Capão do Leão s/n, Pelotas 96010-900, RS, Brazil; andressanornberg@outlook.com.br; 3 NanoBioMat Laboratory, Federal University of Santa Catarina, UFSC, Florianópolis 88040-900, SC, Brazil; m.maraschin@ufsc.br; 4 DNPrime Advanced Ceramics, Içara 88822-856, SC, Brazil; daiara.floriano@gmail.com; 5 Physics Department, Federal University of Santa Catarina, UFSC, Florianopolis 88040-900, SC, Brazil; carlos.campos@ufsc.br; 6 Department of Restorative Dentistry, Federal University of Pelotas (UFPel), Pelotas 96015-560, RS, Brazil; rafael.lund@gmail.com

**Keywords:** bioceramics, hydroxyapatite, bone regeneration, circular economy, osteogenesis

## Abstract

This study aimed to develop a sustainable route for processing biogenic calcium carbonate from *Perna perna* mussel shell waste and converting it into hydroxyapatite (HA), as well as to evaluate its potential for bone and dental tissue engineering applications. Mussel shells were decarbonized (400 °C), milled, and converted to HA via wet chemical precipitation using a nominal Ca/P molar ratio of 1.67 during synthesis followed by thermal treatment (900 °C). Comprehensive characterization included SEM, FTIR, XRD, Raman spectroscopy, XRF, TGA, and BET analysis. Biological evaluation involved cytotoxicity assays (MTT), antimicrobial testing, and odontogenic differentiation studies (Alizarin Red) using SHEDs. Statistical analysis by one-way ANOVA and Tukey post hoc tests (α = 0.05). SEM revealed a microstructured morphology composed of agglomerates, favorable for biomedical applications. FTIR and XRD confirmed the conversion of CaCO_3_ to hydroxyapatite, while thermal analysis demonstrated the material’s stability. The HA exhibited secondary minor phase (13%) β-TCP form of calcium phosphate (Ca_2.997_H_0.006_(PO_4_)_2_), high crystallinity (about 80%), and nanoscale crystallite size (85 nm, 2.5–5.0 m^2^/g), despite forming larger agglomerates in suspension. The material showed favorable physicochemical properties (neutral pH, −18.5 mV zeta potential), but no inhibition was detected in antimicrobial testing. In vitro assays showed excellent cytocompatibility (viability > 70% at 12.5 µg/mL) and significant osteogenic potential (high mineralization vs. controls, *p* < 0.05). Mussel shell-derived HA presents a sustainable, clinically relevant biomaterial with ideal properties for bone regeneration. The study establishes a complete waste-to-biomaterial pipeline while addressing key requirements for dental and orthopedic applications.

## 1. Introduction

The escalating demand for sustainable materials has driven extensive research into the repurposing of biogenic waste. Among these, marine aquaculture-derived shellfish residues, such as oyster, mussel, and seashells, represent a valuable resource for biomedical applications [1,2,3,4,5]. Improper disposal of these residues risks environmental contamination through pest proliferation, disease transmission, and the loss of calcium carbonate (CaCO_3_), a functionally critical component. Consequently, it is imperative to establish more sustainable management practices for these waste streams [6,7,8].

The high value of these residues stems from their potential as a promising source of CaCO_3_ [9]. While their compositions exhibit diverse architectures and microstructures, they primarily consist of CaCO_3_ polymorphs—predominantly aragonite or calcite—encased within an external proteinaceous periostracum [10,11]. Notably, CaCO_3_ nanoparticles demonstrate significant potential in dental applications, including enamel remineralization and dentinal tubule occlusion, which are critical for managing hypersensitivity, incipient caries, and enamel surface loss [12,13,14]. The biocompatibility and abundance of naturally derived CaCO_3_, particularly from mussel and oyster shells, position it as an advantageous alternative for biomedical applications [14,15]. Recent studies have explored the conversion of biogenic CaCO_3_ into calcium phosphates, notably hydroxyapatite [Ca_10_(PO_4_)_6_(OH)_2_], the primary mineral constituent of bones and teeth [16]. Sawada et al. (2021) [17] demonstrated that amorphous calcium carbonate (ACC) can serve as an efficient precursor for pure hydroxyapatite synthesis, emphasizing the role of nanoscale particle size in optimizing this process.

These findings underscore the potential of ACC in biomaterial engineering, with emerging applications in bone regeneration and controlled drug delivery. Synthetic hydroxyapatite remains the most extensively studied calcium phosphate for biomedical uses, including dental implant coatings, drug delivery systems, bioimaging, and orthopedic bone repair. Its widespread adoption is attributed to key properties such as biocompatibility, bioactivity, non-toxicity, and osteointegration [16,18,19]. Hydroxyapatite nanoparticles, in particular, have garnered attention due to their high surface-to-volume ratio, which closely mimics the nanostructure of calcified tissues, thereby enhancing biocompatibility, cellular adhesion, and proliferation [20,21,22,23,24,25,26].

Although several studies have investigated the use of marine shells for hydroxyapatite production, many rely on mixed shell sources or lack detailed biological validation of the final material. This study distinguishes itself by exclusively utilizing Perna perna mussel shells—a specific and abundant waste stream in Southern Brazil—thereby ensuring raw material consistency. Precise control of the processing and conversion steps enabled the production of tailored materials. In addition, we integrated comprehensive physicochemical characterization with a robust biological evaluation using stem cells from human exfoliated deciduous teeth (SHEDs), bridging the gap between material development and potential applications in regenerative dentistry. From a waste valorization perspective, this approach may be considered potentially sustainable; however, a full life cycle assessment was not performed and remains a subject for future investigation.

## 2. Materials and Methods

### 2.1. Preparation of Calcium Carbonate from Mussel Shells

#### 2.1.1. Collection and Pre-Treatment

Only *Perna perna* mussel shells collected from the southern coast of Santa Catarina, Brazil, were used as the calcium source in this study. The shells were immersed in a 2% (*v*/*v*) sodium hypochlorite solution for 24 h at ambient temperature. Initially, all shells were washed with distilled water to remove any surface impurities. To remove remaining organic matter, the shells were soaked in a 2% sodium hypochlorite solution (*v*/*v*) for 24 h at room temperature. Following disinfection, the shells were thoroughly rinsed with deionized water and dried in a convection oven at 100 °C for 48 h. Dried shells were thermally treated in a muffle furnace (Fortelab, São Carlo, SP, Brazil, ME1700) at 400 °C for 2 h to remove any residual organic matter, ensuring a purer inorganic matrix for subsequent processing [24,25,26].

#### 2.1.2. Grinding and Milling

Once cooled to room temperature, the thermally treated shells were crushed in a hammer mill (Servitech, Tubarão, SC, Brazil, CT-12058) to obtain coarse granules. These granules were then subjected to high-energy planetary ball milling (Servitech, Brazil, CT-242) using zirconia jars and balls at 400 rpm for 4 h to produce a fine powder. Milling parameters, including rotation speed, milling time, ball-to-powder ratio, and sieve size, were controlled to ensure reproducibility and uniform particle size distribution prior to subsequent synthesis steps. The final material was passed through a 75 µm mesh sieve to obtain particles with uniform granulometry suitable for characterization and subsequent synthesis.

### 2.2. Synthesis of Hydroxyapatite (HA)

#### Wet Chemical Synthesis

Hydroxyapatite (HA) was synthesized using calcium carbonate powder derived from mussel shells as the calcium source [24,25,26]. A modified wet chemical precipitation method (adapted from da Silva (2019) [24] and Niero et al. (2023) [26]) was employed. Briefly, 100 g of CaCO_3_ powder was gradually dispersed in 250 mL of distilled water under continuous magnetic stirring (Fisatom, São Paulo, Brazil, 713D). Phosphoric acid (85% H_3_PO_4_; Synth) was added dropwise using a peristaltic pump to maintain a constant Ca/P molar ratio of 1.67. The synthesis was carried out at room temperature (22–25 °C) and atmospheric pressure. The peristaltic pump was used exclusively to control the reagent addition rate and did not impose pressurized conditions on the system. The reaction was conducted under controlled laboratory conditions for 40 min, with pH monitored every 20 min to ensure the reaction environment favored hydroxyapatite precipitation. The pH was adjusted using diluted NaOH or HCl to avoid excessive acidity during precipitation, which could affect phosphate incorporation. Although pH monitoring was performed, the subsequent calcination step at 900 °C indicates that the overall process should not be classified as fully biomimetic. After synthesis, the mixture was subjected to attritor milling (Netzsch, Bavaria, Germany, PE 075) for 2 h at 400 rpm in a 500 mL zirconia grinding pot using 3 mm diameter zirconia beads with a ball-to-powder ratio (BPR) of 10:1 to enhance homogeneity and control particle size. The use of zirconia beads and a container minimized potential contamination from the milling process. The obtained suspension was subjected to vacuum filtration through a 0.45 µm cellulose acetate membrane (Millipore Merck KGaA, Darmstadt, Germany) and rinsed three times with deionized water to eliminate soluble byproducts. The filtered material was then dried at 60 °C for 24 h, and manually ground using an agate mortar and pestle to prevent metallic contamination. The powder was sieved through a 150 µm mesh. Calcination was performed at a heating rate of 10 °C·min^−1^ up to 900 °C, followed by a dwell time of 2 h, in a muffle furnace (Fortelab, QR-1300/3E). These conditions were selected based on thermal analysis results to promote phase stabilization and removal of residual volatile species.

### 2.3. Morphological Characterization of CaCO_3_ Powder and Synthesized HA

#### Scanning Electron Microscopy (SEM)

The morphological characterization of the powders was performed using scanning electron microscopy (SEM). The samples were first suspended in ethanol and exposed to ultrasonic agitation for 1 min to achieve uniform dispersion. A small portion of this suspension was then deposited onto metallic stubs that had been pre-coated with double-sided carbon tape. After drying at room temperature, the samples were sputter-coated with a gold–palladium alloy (thickness between 300 Å and 500 Å) for 120 s to enhance conductivity. SEM analysis was conducted using a JEOL JSM-6390LV microscope (Tokyo, Japan) operated at accelerating voltages of 10 and 20 kV [26].

### 2.4. Physicochemical Characterization of Synthesized Materials

#### 2.4.1. Physicochemical Characterization of CaCO_3_ Powder and Synthesized HA

Fourier Transform Infrared Spectroscopy (FTIR) analyses were conducted using a Spectrum Two FT-IR Spectrometer (Spectrum Two—Perkin Elmer, Shelton, CT, USA), using a spectral range of 4000–550 cm^−1^ and a resolution of 4 cm^−1^, roughly 1 mg of the sample was mixed thoroughly with 100 mg of KBr and pressed into pellets for transmission analysis [26].

#### 2.4.2. Thermal Analysis

Thermal behavior and decomposition profiles of the synthesized CaCO_3_ and HA were evaluated using simultaneous thermogravimetric and differential scanning calorimetry (TG/DSC) on an SDT-Q600 instrument (TA Instruments, New Castle, DE, USA). Analyses were performed from ambient temperature up to 850 °C at a steady heating rate of 10 °C/min under a nitrogen atmosphere [24].

#### 2.4.3. X-Ray Diffraction (XRD)

X-ray diffraction (XRD) was carried out using an Xpert PANalytical powder X-ray Diffractometer (Malvern PANalytical, Worcestershire, United Kingdom) with kα–Cu radiation in the 2θ angle range from 3 to 90°, equipped with an X’Celerator detector and using a Ni filtered radiation of Cu Kα (λ = 1.5418 Å), the tension and current used were 45 kV and 40 mA, respectively., At least three batches (#1, #2, #3 and #4) of each CaCO_3_ and HA powder samples were analyzed in the 2θ angle range from 3 to 90° (once up to 152° to one of the HA samples), with 0.05° stepsize and 120 s counting time with at least 3 scans averaged to improve data statistics, totalizing about 130 min of data collection per sample. The samples (CaCO_3_ #1, #2 and HA #3) were prepared in large-volume stainless steel sample holders (diameter of 27 mm and depth of 2.4 mm), while the samples (CaCO_3_ #3 and HA #3) were prepared inside a small cavity with diameter of 10 mm and depth of 0.2 mm on Silicon ZeroBackground (ZB) plates.

The identification of peaks in the XRD patterns was performed using the PANalytical X’Pert HighScore 2.2 software, and the Powder Diffraction Files (PDF) from ICDD (International Centre for Diffraction Data). The crystallographic Information File (CIF) from Inorganic Crystal Structure Database (ICSD) was used in the initial modeling in Rietveld analysis. XRD data analysis to obtain the structural and microstructural information was performed using the Rietveld Method implemented in the TOPAS software package (version 6) [27]. The phase fractions by weight were determined through the quantitative phase analysis available in the same software. For XRPD line profile evaluation and microstructural characterization, Fundamental Parameters Approach (FPA) was applied using the standard double-Voigt (DV) method. The quality of the adjustments was monitored by the agreement parameters R-weighted profile Rwp and Goodness of Fit GOF.

#### 2.4.4. Raman Spectroscopic Analysis

Raman spectra of CaCO_3_ and HA powders were recorded using a PeakSeeker PRO-785 Raman spectrometer (Agiltron, Woburn, MA, USA) equipped with a 50× objective lens in backscattering geometry at ambient temperature. A diode laser with a wavelength of 785 nm and a power of 50 mW at the source was employed. Spectra were acquired at a resolution of 6 cm^−1^ over the 200–2000 cm^−1^ spectral range using a Peltier-cooled charge-coupled device (CCD detector). Additionally, spectra were collected with a 532 nm excitation laser at 25 mW using a Via Raman system using a 20× objective lens at ambient temperature [27]. The powder samples were prepared on glass slides coated with aluminum foil (using a flat spatula to make the powder surface as flat as possible) and placed on the stage of the optical microscope of the Raman systems used.

#### 2.4.5. X-Ray Fluorescence Spectroscopic Analysis (XRF)

The chemical composition of CaCO_3_ was analyzed using a Bruker S2 Ranger wavelength-dispersive XRF spectrometer (Bruker AXS GmbH, Karlsruhe, BW, Germany). Samples were dried at 150 °C for 40 min, then 1 g of the sample was mixed with 10 g of lithium tetraborate. The mixture was fused at 950–1000 °C and cast into a glass disk. Major oxides quantified included SiO_2_, Al_2_O_3_, Fe_2_O_3_, CaO, Na_2_O, K_2_O, MnO, TiO_2_, MgO, and P_2_O_5_.

#### 2.4.6. Nanoscale Morphology and Density Characterization

The zeta potential (ζ) of the samples was determined using a Zetasizer Nano Zen3600 (Malvern Panalytical, Worcestershire, UK) in aqueous HEPES buffer (25 mM, pH 7.2) with a particle concentration of 0.1 mg mL^−1^. The pH was adjusted as needed with 1 M NaOH or HCl. Porosity was assessed through helium pycnometry (Ultrapyc 1200e, Quantachrome, Boynton Beach, FL, USA), with total porosity calculated by comparing the apparent and true densities of the material. To assess fluid absorption capacity, approximately 50 mg of the powdered sample was immersed in simulated body fluid (SBF) at 37 °C for 24 h. The percentage of fluid uptake was calculated from the difference in pre- and post-immersion weights.

The specific surface area of HA powders was measured using the Brunauer–Emmett–Teller (BET) method on a NOVA 1200e analyzer (Quantachrome). Samples were degassed at 150 °C for 12 h before nitrogen adsorption measurements. The pH of a 10% (*w*/*v*) aqueous suspension of HA was measured at room temperature using a calibrated digital pH meter. Oxide residue content (R_2_O_3_) was quantified by inductively coupled plasma optical emission spectrometry (ICP-OES), targeting residual trivalent metal oxides such as Al_2_O_3_ and Fe_2_O_3_. The density of the HA powder was determined using a helium gas pycnometer.

### 2.5. Biological Evaluation of CaCO_3_ Powder and Synthesized HA for Biomedical Applications

#### 2.5.1. Antimicrobial Activity Assay

The antimicrobial activity of CaCO_3_ powder and synthesized HA was evaluated by determining their minimum inhibitory concentrations (MICs) against selected bacterial and fungal strains. The tested concentrations were 6.25, 12.5, 25, 50, and 100 µg/mL (*n* = 4 for each concentration and material). MIC determination was based on the broth microdilution method, adapted from M60 (CLSI, 2020) [28] and M07 reference documents (CLSI, 2018) [29], using 96-well microtiter plates.

The microbial strains used included *Enterococcus faecalis* (ATCC 51299), *Streptococcus mutans* (ATCC UA159), *Staphylococcus aureus* (ATCC 19095), and *Candida albicans* (ATCC 10231). Bacterial strains were cultured in Brain Heart Infusion Broth (BHI; Kasvi, São José dos Pinhais, PR, Brazil), and yeast strains in Sabouraud Dextrose Broth (SDB; Kasvi, São José dos Pinhais, PR, Brazil). Reactivated microorganisms were incubated at 37 °C for 24 h. Standardized inocula were prepared by adjusting microbial suspensions to a 0.5 McFarland standard (10^8^ CFU/mL for bacteria and 10^6^ CFU/mL for yeasts). These suspensions were subsequently diluted 1:10 in their respective culture media to obtain a final inoculum of approximately 10^7^ CFU/mL for bacteria and 10^5^ CFU/mL for fungi.

Each well received 100 µL of the respective powder diluted in the appropriate culture medium at the tested concentrations, along with 5 µL of the microbial inoculum. Positive controls consisted of culture medium and inoculum without the tested powders, while negative controls contained only the culture medium and powder (without inoculum) to confirm sterility. Plates were incubated at 37 °C for 24 h under aerobic conditions.

After incubation, microbial growth was visually assessed based on the turbidity of the medium. Wells showing no visible growth were considered inhibitory. To confirm the microbicidal effect, 20 µL of each clear well was replated on solid BHI agar (for bacteria) or Sabouraud Dextrose Agar (for fungi) and incubated under the same conditions for 24 h. Absence of growth confirmed bactericidal or fungicidal activity. The MIC was defined as the lowest concentration of each powder at which no visible microbial growth was observed.

#### 2.5.2. Cytotoxicity Assay

To evaluate the cytotoxic potential of the obtained CaCO_3_ powder and the synthesized HA. Stem cells derived from human-exfoliated deciduous teeth (SHEDs) were supplied by Curityba Biotech^®^ Cell Processing Center (Curitiba, PR, Brazil). SHEDs were exposed to different concentrations of each material: 6.25, 12.5, 25, 50, and 100 µg/mL (n = 6). The powder samples were sterilized using ultraviolet light and suspended in high-glucose DMEM (GIBCO, Thermo Fisher Scientific Inc., Waltham, MA, USA), supplemented with 10% fetal bovine serum (FBS, GIBCO).

SHEDs at passage 5 were cultured in 96-well plates at a density of 2 × 10^4^ cells per well and incubated for 24 h at 37 °C in a humidified atmosphere with 5% CO_2_ to promote cell adhesion. Afterward, the culture medium was replaced with fresh medium containing the respective concentrations of CaCO_3_ or HA. Cell viability was assessed after 24 h of exposure using a colorimetric thiazolyl blue tetrazolium bromide (MTT, Sigma-Aldrich, Burlington, MA, USA) assay.

For the MTT assay, 10 µL of MTT solution (5 mg/mL) was added to each well and incubated for 4 h at 37 °C. Formazan crystals formed by metabolically active cells were then dissolved with 100 µL of dimethyl sulfoxide (DMSO), and absorbance was measured at 540 nm using a microplate reader (Infinite M200, Tecan Group Ltd., Männedorf, ZH, Switzerland). Cell viability was expressed as a percentage relative to the untreated control group. According to ISO 10993-5 guidelines, values below 70% were considered indicative of cytotoxicity [30,31].

#### 2.5.3. Odontogenic Differentiation Assays

CaCO_3_ powder and synthesized HA were sterilized using were sterilized by ultraviolet light. SHEDs at passage 5 were seeded at a density of 3 × 10^4^ cells per sample for odontogenic differentiation, which was evaluated by Alizarin Red Staining (ARS). SHEDs cultured in basal medium served as the negative control, while those maintained in odontogenic differentiation medium—supplemented with Dulbecco’s Modified Eagles’ Medium (DMEM; Gibco, Thermo Fisher Scientific, Waltham, MA, USA) with 10% fetal bovine serum (FBS; Gibco, Thermo Fisher Scientific, Waltham, MA, USA), 50-µM L-ascorbic acid (ASC; A4544, Merck KGaA, St. Louis, MO, USA), 10 mM βGLY (G9891, Merck KGaA, St. Louis, MO, USA), 0 μg/mL ascorbic acid, and 10 mM β-glycerophosphate for subculturing at 37 °C with an atmosphere of 5% of CO_2_—were used as the positive control. Cells were seeded in 24-well plates, and scaffolds were mounted in sterile plastic transwell inserts to investigate the effect of β-tricalcium phosphate release on odontogenic differentiation using ARS assay. ARS was on day 14.

For ARS (ScienCell Research Laboratories, Carlsbad, CA, USA), at the designated time points, culture medium was removed, and cells were rinsed three times for 15 min at room temperature with PBS, then fixed with 4% formaldehyde for 15 min. After fixation, cells were washed with ultra-pure water and stained with 150 µL of 3% ARS solution per well for 30 min, protected from light. Bound ARS was subsequently solubilized with 150 µL of 5% SDS in 0.5 N HCl for 30 min. Absorbance was measured at 595 nm [31].

### 2.6. Statistical Analysis

Data analysis was conducted using SPSS 21.0 (SPSS Inc., Chicago, IL, USA), considering a 95% confidence interval. Normality was evaluated using the Shapiro–Wilk test, while homogeneity of variances was assessed with the Levene test. Following these assumptions, cellular viability from the MTT and ARS assays was analyzed at a significance level of α = 0.05 using one-way ANOVA (ordinary), followed by Tukey’s multiple comparisons test for post hoc analysis.

## 3. Results

### 3.1. Morphological Characterization of CaCO_3_ Powder and Synthesized HA

#### Scanning Electron Microscopy (SEM)

**Figure 1:**
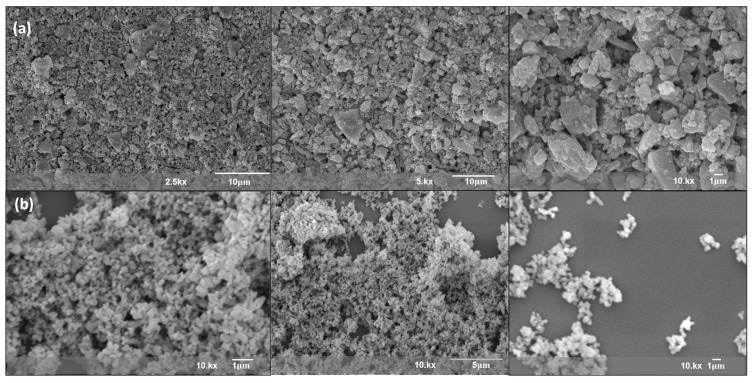
SEM micrographs of (**a**) CaCO_3_ powders derived from Perna perna mussel shells and (**b**) hydroxyapatite (HA) obtained after conversion, shown at increasing magnifications.

SEM images revealed agglomerated particles with irregular morphology, which is commonly reported for hydroxyapatite powders synthesized via wet precipitation routes [32]. Due to agglomeration and resolution limits, no definitive particle shape could be assigned solely based on SEM analysis (Figure 1). The shell powder (Figure 1a) shows irregular, angular particles, while the HA (Figure 1b) displays sub-micron morphology with agglomerated, spherical-like particles. While individual crystallites may be nanoscale, the SEM images show significant agglomeration, typical of hydroxyapatite powders. The particles are unevenly distributed, with a tendency to form clusters, which is typical of materials synthesized through biomimetic routes. The scale features and high surface area observed are favorable for biomedical applications that require enhanced surface reactivity and cellular interaction. SEM images provide qualitative information on particle morphology and agglomeration behavior but do not allow quantitative assessment of surface area or direct inference of biological performance.

### 3.2. Physicochemical Characterization of Synthesized Materials

#### 3.2.1. X-Ray Fluorescence Spectroscopic Analysis (XRF)

**Figure 2:**
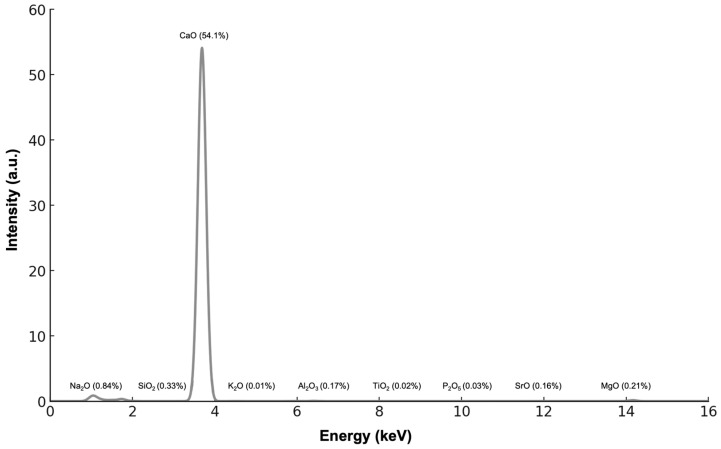
X-ray fluorescence (XRF) results and elemental oxide composition of CaCO_3_ derived from mussel shells. Values are expressed as weight percentage (wt%) of oxides.

Figure 2 shows x-ray fluorescence (XRF) results and percentage of oxide composition of CaCO_3_ derived from mussel shells. Calcium oxide (CaO) was the predominant component (54.1 wt%). It is important to note that XRF provides elemental composition expressed as oxides, which does not imply the presence of free CaO phase, but rather confirms the high calcium content of the carbonate matrix. Minor oxides included silicon dioxide (SiO_2_, 0.33 wt%), sodium oxide (Na_2_O, 0.84 wt%), magnesium oxide (MgO, 0.21 wt%), and phosphorus pentoxide (P_2_O_5_, 0.03 wt%). Trace amounts of aluminum oxide (Al_2_O_3_, 0.17 wt%), potassium oxide (K_2_O, 0.01 wt%), titanium dioxide (TiO_2_, 0.02 wt%), and strontium oxide (SrO, 0.16 wt%) were also present. Iron oxide (Fe_2_O_3_) and manganese oxide (MnO) were not detected in the analyzed sample. XRF analysis was not performed on HA samples due to the limited sample mass available and because phase identification and structural analysis were primarily addressed by XRD.

#### 3.2.2. X-Ray Diffraction (XRD)

**Figure 3:**
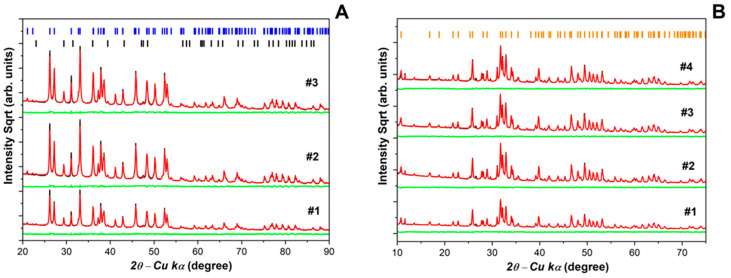
Experimental and calculated X-ray diffraction patterns of (**A**) three batches (#) of the CaCO_3_ powders derived from mussel shells, showing the tickmarks corresponding to aragonite (blue bars) and calcite CaCO_3_ (black bars) phases (Rwps from 7.64 up to 7.78 and GOFs from 3.28 up to 5.08), and (**B**) four batches (#) of the synthesized nanohydroxyapatite (HA) (Rwp 5.14–6.82 and GOF 2.84–4.27) with tickmarks of HA phase (orange bars). Red lines correspond to experimental data, black lines to calculated patterns, and green lines represent the difference between experimental and calculated patterns, which, together with Rwp and GOF values, indicate an acceptable agreement between experimental and calculated patterns.

The X-ray diffraction measurements attest to the reproducibility of the sample preparation for all batches analyzed as is shown in Figure 3. Figure 3A shows the Rietveld analysis of the diffractograms obtained for the CaCO_3_ powder samples. Multiple synthesis batches were analyzed to verify phase reproducibility. No significant differences were observed among batches, and representative diffractograms are therefore presented. The structural and microstructural information of the samples was determined. The major crystalline counterpart (93 wt%) of the sample is from the orthorhombic CaCO_3_ aragonite phase (*Pmcn* space group, ICSD-32100), while the complementary crystalline portion (7 wt%) was attributed to the rhombohedral CaCO_3_ calcite phase. Preferential Orientation modeling in directions (002) and (012) was needed to properly describe the experimental pattern of the aragonite phase. In the HA diffractograms, Figure 3B, the most intense peaks correspond to the hexagonal hydroxyapatite phase (Ca_5_(PO_4_)_2_(OH); *P63/m* space group, ICSD-22060), while at about 11–13 wt% was identified as β-TCP form of calcium phosphate (Ca_2.997_H_0.006_(PO_4_)_2_), rhombohedral R3cH space group, ICSD-6191, which mineral name is whitlockite). All secondary phases are indicated in the figure. One relatively intense Bragg peak can be seen at about 11.7o. It was associated with monoclinic Ca(HPO4) (H_2_O)_2_ phase (Ia space group, ICSD-16132, mineral name brushite), which is a minor phase (<0.5 wt%) for most of the batches analyzed. Moreover, a typical non-crystalline halo can be observed at the low-angle region (<20°), and it was used to estimate the sample crystallinity in TOPAS (Rietveld analysis) at about 75%. This value is in reasonable agreement with the 83% obtained by using the intensities of (300) reflection and the valley between (112) and (300) reflections (named V112/300 Landi et al.) [33]. Crystallinity was estimated using both Rietveld refinement and the V112/300 method described by Landi et al. [33], based on peak intensity ratios. The microstructural characterization of the hydroxyapatite phase showed an average crystallite size of approximately 83 nm with a microstrain of 0.15. Quantitative Rietveld refinement was performed on representative batches due to the high reproducibility observed across syntheses, with variations remaining within instrumental uncertainty. See Table 1 for more results of the XRD–Rietveld analyses.

#### 3.2.3. Raman and FTIR Spectroscopic Analyses

**Figure 4:**
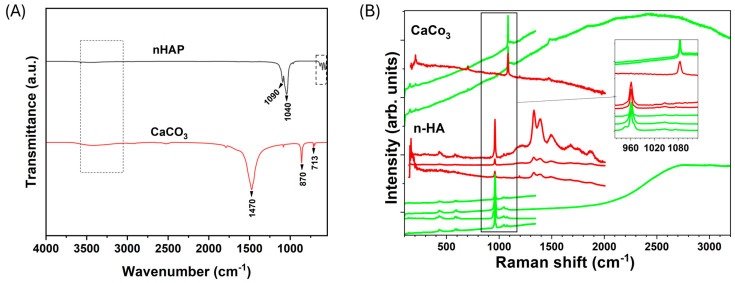
(**A**) FTIR and (**B**) Raman spectra (transmittance mode) of mussel shell powder (CaCO_3_) and synthesized HA. Red and green lines in (**B**) correspond to the Raman spectra collected using red (@785 nm) and green (@532 nm) laser excitations, respectively.

Characteristic carbonate bands are observed in the shell (CaCO_3_) powder, while HA shows phosphate (PO_4_^3−^) bands and hydroxyl group absorptions typical of hydroxyapatite. The FTIR spectrum of the mussel shell (CaCO_3_) powder (Figure 4A) revealed characteristic absorption bands of calcium carbonate. A strong asymmetric stretching vibration of carbonate (ν_3_) was observed around 1400–1470 cm^−1^, while bending modes (ν_2_ and ν_4_) were identified at approximately 870 cm^−1^ and 710–730 cm^−1^, respectively. Broad absorption bands near 3400 cm^−1^ and 1600 cm^−1^ indicated the presence of adsorbed water. These results confirm the predominance of CaCO_3_ in the shell matrix, with spectral features corresponding to calcite and aragonite polymorphs. For example, a broad band at 3400 cm^−1^ is attributed to the OH stretching of the adsorbed water, a band at 1470 cm^−1^ is associated with the asymmetric C–O stretching of the carbonate anion (CO_3_^2−^), bands at 870 and 713 cm^−1^ are attributed to the bending modes of calcite (CaCO_3_ polymorphs) [34,35]. The additional broad bands observed between 1100 and 1700 cm^−1^ under 785 nm excitation may be associated with fluorescence effects, structural disorder, or residual organic traces, which are commonly reported for biogenic-derived materials and do not correspond to phosphate vibrational modes. From these results, it is possible to confirm the predominance of CaCO_3_ in the shell matrix, with spectral characteristics corresponding to the calcite and aragonite polymorphs. The Raman spectra of mussel shell (CaCO_3_) powder (Figure 4B) exhibited complimentary picture with a strong peak at ~1085 cm^−1^, corresponding to the symmetric stretching mode (ν_1_) of the carbonate group (CO_3_^2−^), along with minor peaks at ~150 cm^−1^, 203 cm^−1^, 700 cm^−1^ and ~1470 cm^−1^, associated with lattice vibrations and in-plane bending, respectively. These peaks cannot distinguish between carbonate phases (aragonite and calcite), once both phases have these features (see R040078-1 for aragonite and R050128 cards at RUFF database). No baseline treatment was performed in order to highlight photoluminescent effects according to the laser excitation applied in each experiment.

In contrast, the FTIR spectrum of the synthesized HA (Figure 4A) revealed bands attributed to phosphate (PO_4_^3−^) groups, including intense absorptions at ~1090 and ~1030 cm^−1^ (ν_3_), ~960 cm^−1^ (ν_1_), and ~602 and ~570 cm^−1^ (ν_4_), as well as a band at ~470 cm^−1^ (ν_2_). These features can be associated with the characteristic bands of the phosphate group (PO4-3), including the bands around 1090, 1040, 962, and 600–560 cm^−1^ that are associated with the vibrations of the PO_3_, P–O–P, and PO_4_^−3^ bands [36,37]. Additionally, the presence of hydroxyl groups was confirmed by bands at ~3570 cm^−1^ (stretching mode) and ~630 cm^−1^ (vibration mode), which are characteristic of hydroxyapatite. In addition, carbonate substitutions were evaluated by means of shoulder-type bands in the regions of 1410–1470 cm^−1^ and ~870 cm^−1^, indicating B-type carbonated hydroxyapatite. Complementary to the Raman spectra of the synthesized HA (Figure 4B) displayed most intense peak at about ~960 cm^−1^ with both green and red excitations. A closer look at this feature (measured with green laser, see inset in Figure 4B) revealed a very intense peak at 961 cm^−1^ with two shoulders, at 946 cm^−1^ and 968 cm^−1^. The most intense can be attributed to the symmetric stretching mode of the phosphate group (ν_1_ PO_4_^3−^) of hydroxyapatite, while both satellite peaks can be associated with PO_4_ vibration lines of the β-TCP form of calcium phosphate (Ca_2.997_H_0.006_(PO_4_)_2_), whitlockite), in close connection with XRD results [38]. Small overlapped peaks are observed at about 400 cm^−1^, 600 cm^−1^ and 1050 cm^−1^ (see yellow inset in Figure 4B), which can also be attributed to ν_n_ PO_4_^3−^ modes of hydroxyapatite, confirming the formation of hydroxyapatite. However, extra features along with at least four additional broad bands between 1100 and 1700 cm^−1^ were only observed for @785 nm excitation, not assigned to phosphate (hydroxyapatite) vibrational modes nor to β-TCP form of calcium phosphate (whitlockite) ones. No carbonate peaks were detected in the HA sample.

#### 3.2.4. Thermal Analysis

**Figure 5:**
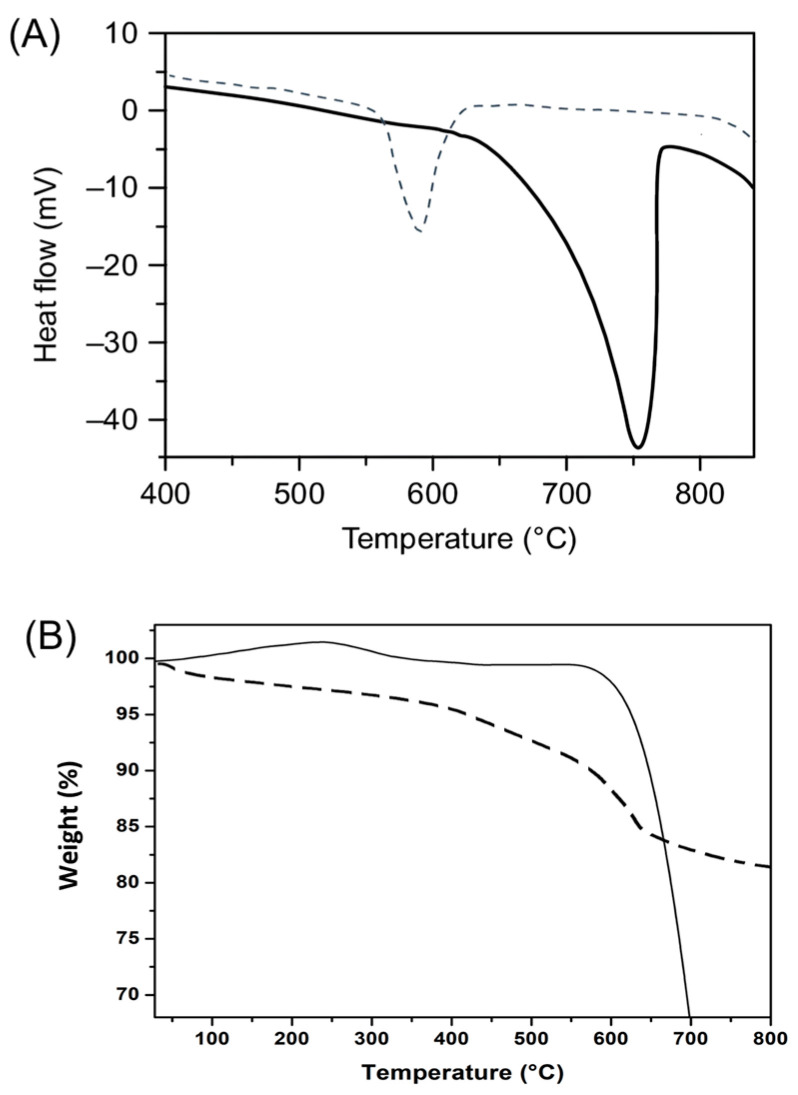
Thermal analysis of CaCO_3_ and synthesized nanohydroxyapatite (HA). (**A**) Differential Scanning Calorimetry (DSC): The CaCO_3_ sample shows an intense endothermic peak near 740 °C, consistent with its decomposition into CaO and CO_2_. The HA (dotted line) displays a broad endothermic signal around 150–180 °C, attributed to the release of adsorbed or structural water. (**B**) Thermogravimetric Analysis (TGA): CaCO_3_ exhibits a sharp mass loss above 700 °C (interruption in the line is due to the limitation in the data collection time, with no measurements taken beyond this temperature), confirming thermal decomposition. The HA (dotted line) shows gradual weight loss up to 800 °C, associated with moisture loss and structural water release, indicating good thermal stability.

The thermal behavior of mussel shell powder and synthesized nanohydroxyapatite (HA) was evaluated using Differential Scanning Calorimetry (DSC) and Thermogravimetric Analysis (TGA), as shown in Figure 5.

The DSC curve of mussel shell powder (Figure 5A) indicates that thermal decomposition began at a slow rate and underwent rapid recomposition with increasing temperature, revealing a sharp endothermic peak at approximately 740 °C, attributed to the thermal decomposition of calcium carbonate into calcium oxide (CaO) and carbon dioxide (CO_2_). In contrast, the DSC curve of HA (Figure 5A-dotted line) showed a broad endothermic event between 150 and 180 °C, probably due to the loss of adsorbed or structural water molecules from the hydroxyapatite structure.

In the TGA, the mussel shell powder sample (Figure 5B showed a pronounced mass loss from approximately 700 °C, attributed to the loss of gaseous carbon dioxide (CO_2_) emission, confirming its thermal degradation [37]. On the other hand, the HA sample (Figure 5B-dotted line) showed a more gradual and continuous mass loss throughout the heating range up to 800 °C. For example, up to 200 °C, moisture is removed from the sample, after which, in the range of 250 to 600 °C, the decomposition of the HPO_4_^2−^ portion occurs and the removal of interstitial water present in the chemical structure of HA, and finally, there is a decomposition in the range of 650–850 °C, with the final decomposition of the P_2_O_7_^4−^ portion [39,40]. That is, this behavior is consistent with the loss of water, both from surface moisture and structurally bound water, without a defined decomposition step, suggesting that the synthesized HA has greater thermal stability.

#### 3.2.5. Nanoscale Refinement and Characterization

Table 2 shows zeta potential, particle size (Z-Ave), electrophoretic mobility, conductivity, and polydispersity index (PdI) measurements of CaCO_3_ and HA particles. The physicochemical characterization of HA and CaCO_3_ revealed distinct properties. HA exhibited a zeta potential of −18.47 ± 1.09 mV, a particle size (Z-Ave) of 179.73 ± 20.24 nm, and a polydispersity index (Pdl) of 0.744 ± 0.074. In contrast, CaCO_3_ showed a zeta potential of −18.00 ± 0.40 mV, a larger particle size (410.50 ± 17.04 nm), and a lower Pdl (0.603 ± 0.150). Both particles displayed similar electrophoretic mobility (for HA and for CaCO_3_) but differed in conductivity (HA: 0.02543 mS/cm; CaCO_3_: 0.05783 mS/cm).

Further analysis of HA (Table 3) indicated high crystallinity (75–83%), purity (93%), and a crystallite size of ~60 nm. The pH of a 10% HA solution ranged between 7.0 and 8.0, with a surface area of 2.5−5.0 m^2^/g. HAP CF C900 content reached 1004.62 wt%. The density of the synthesized HA ranged from 3.09 to 3.30 g/cm^3^, indicating a compact and dense structure consistent with high-quality hydroxyapatite.

### 3.3. Biological Evaluation of CaCO_3_ and HA Particles for Biomedical Applications

#### 3.3.1. Antimicrobial Activity Assay

Table 4 shows minimum inhibitory concentrations (MIC, mg/mL) determined for CaCO_3_ powder and synthesized HA particles against *E. faecalis*, *S. mutans*, *S. aureus*, and *C. albicans*. No antimicrobial effect was observed for either material.

#### 3.3.2. Cell Viability

**Figure 6:**
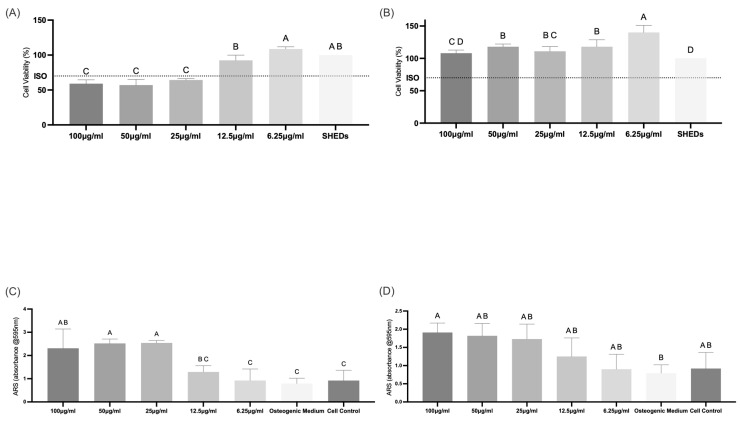
Cytotoxicity and osteogenic potential of CaCO_3_ and HA on SHEDs. (**A**,**B**) Cell viability was assessed by MTT assay after 24 h. CaCO_3_ (**A**) maintained viability > 80% at higher concentrations (50–100 µg/mL), while HA (**B**) showed optimal viability at the lowest concentration (6.25 µg/mL). (**C**,**D**) Extracellular mineralization via Alizarin Red S staining after 14 days. CaCO_3_ (**C**) enhanced mineralization at 25–100 µg/mL, whereas HA (**D**) exhibited peak mineralization at 6.25 µg/mL. Data presented as mean ± SD. Uppercase letters indicate statistical differences (*p* < 0.05).

The cytotoxic effects of CaCO_3_ and HA extracts were evaluated using the MTT assay after 24 h of exposure on stem cells from human exfoliated deciduous teeth (SHEDs). The materials demonstrated distinct biological responses depending on concentration. For CaCO_3_ (Figure 6A), cell viability remained above the 70% threshold established by ISO 10993-5 [30] across all tested concentrations. The SHED control group exhibited high viability, statistically comparable to that of the 100 µg/mL group. Conversely, for HA (Figure 6B), lower concentrations (12.5 and 6.25 µg/mL) maintained viability above 70% with significant differences between them. Notably, the lowest concentration (6.25 µg/mL) presented the highest cell viability.

#### 3.3.3. Extracellular Mineralization

Osteogenic differentiation was evaluated via Alizarin Red S (ARS) staining after 24 h of culture. For CaCO_3_ (Figure 6C), significant mineralized nodule formation was observed at concentrations of 25, 50, and 100 µg/mL, with absorbance values surpassing both negative (basal medium) and positive (osteogenic medium) controls (*p* < 0.05), indicating a stimulatory effect on differentiation. Lower concentrations (12.5 and 6.25 µg/mL) produced intermediate levels of mineralization, statistically comparable to the controls.

In the case of HA, the 6.25 µg/mL concentration stood out showing mineralization levels comparable to other concentrations and to the positive control, according to the statistical groupings presented in Figure 6D (*p* < 0.05). Although other concentrations showed absorbance similar to the positive control, no group exhibited an inhibitory effect on mineralization.

## 4. Discussion

SEM analysis indicated that the process yielded a micro/nanostructured material. The observed structure exhibited a porous arrangement, and the particles tended to agglomerate. While density measurements indicate a compact bulk material, the surface morphology retains roughness favorable for biological interactions [6,8,14,18,22,25,26]. The observed structure exhibited a porous arrangement, with particles forming larger plate-like assemblies—a common behavior in biogenic-derived hydroxyapatites due to their high surface energy and reactivity [16,24,40]. While the equipment resolution limited the detection of isolated nanoparticles, the prevalent agglomeration in the samples aligns with the expected tendency of such materials. Importantly, while density measurements indicate a compact bulk structure, SEM observations reflect particle-level agglomeration rather than intrinsic porosity. These parameters describe different structural scales and are not contradictory [4].

The XRD analysis confirmed that the synthesis process effectively promotes the phase transformation from calcium carbonates (CaCO_3_) to nanohydroxyapatite (HA). The diffraction pattern of the CaCO_3_ precursor revealed sharp peaks characteristic of CaCO_3_ aragonite and calcite (<7%), indicating high crystallinity prior to conversion, similar to that observed for eggshells [22,41]. After the synthesis process, the XRD pattern of the resulting material exhibited biphasic HA/β-TCP with 86% HA Ca_5_(PO_4_)_2_(OH) phase and 13% of β-TCP form of calcium phosphate (Ca_2_._997_H_0.006_(PO_4_)_2_). This outcome is related to the choice of phosphoric acid as a precursor and the thermal treatment at 900 °C, both of which promote the partial formation of β-TCP. The coexistence of HA and β-TCP may be beneficial for biomedical applications, as β-TCP is more resorbable and enhances calcium and phosphate ion release, stimulating osteogenic responses.

The biphasic composition is advantageous because, while HA ensures long-term chemical stability and biocompatibility, its limited resorption can hinder full integration with native bone [22,40]. The presence of β-TCP introduces a more soluble component that gradually dissolves, releasing calcium and phosphate ions into the microenvironment. This process supports osteogenic cell activity and accelerates bone remodeling compared to monophasic HA [5,39,41]. Also, HA provides long-term stability, while β-TCP dissolves faster and releases ions that promote bone regeneration. The ~13% β-TCP fraction may offer a balanced resorption profile, but dissolution and ion-release studies in physiological-like environments are required to validate in vivo performance and correlate kinetics with osteoconductive outcomes [8,16,40]. This HA/β-TCP systems were extensively reported by authors who used cuttlefish bones, mussel shells, chicken eggshells, and bioinspired amorphous calcium carbonate to synthesize hydroxyapatite nano-powders [2,22,40,41]. Importantly, in contrast to previous studies that predominantly used conventional phosphate sources such as Na_2_HPO_4_ and (NH_4_)_2_HPO_4_ [1,2,3,4,5,40], our method utilized a new phosphate source, which has been rarely reported in the literature for HA synthesis [24,25,26]. This novel choice of phosphate precursor may have contributed to the controlled formation of the biphasic HA/β-TCP system and influenced both the crystallinity and morphology of the synthesized material, suggesting a degree of tunability through precursor selection [40]. The high crystallinity of the HA phase is particularly significant, as it is known to enhance the structural integrity and long-term stability of bioceramic scaffolds. Crystalline hydroxyapatite is less prone to resorption and more capable of maintaining its architecture during bone remodeling, compared to its poorly crystalline counterparts [4,8,16].

The broadening of the diffraction peaks in the HA sample was modeled using the FPA in XRD-Rietveld analyses, indicating the formation of hydroxyapatite with crystallite size domains in the nanoscale range (average ~83 nm). XRD estimates the size of individual crystalline domains, whereas DLS measures the hydrodynamic diameter of particle agglomerates in suspension. Therefore, the larger Z-Ave values reflect aggregation of multiple crystallites rather than discrepancies between techniques. The slightly larger Z-Ave value suggests that these crystallites tend to form small aggregates in aqueous media, which is a well-documented behavior of hydroxyapatite due to its high surface energy. The structural purity and thermal stability confirmed by XRD and DSC support the suitability of the synthesized HA for further investigation in hard tissue-related applications [20,21,24,25,36,41]. Residual carbonate groups may substitute either hydroxyl (A-type) or phosphate (B-type) sites in the hydroxyapatite lattice, which is commonly reported for biogenic-derived HA. Such carbonate substitution can destabilize the HA structure at elevated temperatures, contributing to the partial formation of β-TCP observed after thermal treatment at 900 °C. Thermal annealing promoted improved crystallinity, as evidenced by peak sharpening in XRD patterns; further optimization may be achieved by adjusting heating rate and dwell time.

The Raman and FTIR analyses provide clear evidence of the successful structural transformation from calcium carbonates to hydroxyapatite. The spectrum of the raw shell powder showed well-defined bands typical of calcite, the most stable polymorph of CaCO_3_ [24]. The prominent carbonate vibrations observed are consistent with previous reports on marine biogenic carbonates, supporting the identification of the precursor material [1,2,3,4,5,6,7,8,9]. The broad O–H band suggests moisture retention, and the presence of a minor C–H stretching band near 2920 cm^−1^ points to traces of organic compounds [16]. The disappearance of characteristic carbonate bands and the emergence of well-defined phosphate signals—especially the prominent ~960 cm^−1^ band—demonstrate the formation of a phosphate-rich hydroxyapatite phase [19]. The presence of multiple phosphate-associated bands further supports the formation of a biphasic HA/β-TCP system [2,22,40,41]. However, further studies are in progress to elucidate such extra features observed in the Raman spectrum of the HA sample using 785 nm laser excitation [19]. These results validate the chemical conversion route used for synthesis and confirm that the resulting material possesses the spectral signature of nanohydroxyapatite. This transformation is critical for biomedical applications, as the phosphate-rich surface of HA is known to support bioactivity and osteoconductivity, distinguishing it from carbonate-based precursors [19].

The thermogravimetric data provided further insight into the compositional purity and structural characteristics of both the precursor and synthesized nanomaterial. The sharp weight loss observed in the CaCO_3_ sample at high temperatures is typical of calcite and confirms the absence of significant secondary phases [14,24]. The thermal profile of HA, with its broad, lower-magnitude losses, is consistent with well-crystallized hydroxyapatite containing trace moisture and negligible organic contamination. Importantly, the absence of a distinct carbonate decomposition step in the HA curve supports the FTIR findings of minimal carbonate retention, indicating a complete transformation of CaCO_3_ to stoichiometric Ca_10_(PO_4_)_6_(OH)_2_. Notably, significant thermal events in HA are typically observed above 600 °C, reflecting structural reorganization or minor phase transitions associated with the hydroxyl and phosphate groups, which is consistent with the behavior of pure and thermally stable hydroxyapatite. This thermal behavior further corroborates the efficiency of the biomimetic synthesis route and confirms the thermal resilience of the final HA product for biomedical use [2,24].

The zeta potential values of HA and CaCO_3_ suggest moderate colloidal stability, which is critical for biomedical applications where particle aggregation must be minimized. The larger particle size of CaCO_3_ compared to HA may influence its interaction with biological systems, potentially affecting cellular uptake and degradation rates. The higher Pdl of HA indicates a broader size distribution, which could impact its mechanical properties when used in composite biomaterials [16,22,24].

It is important to highlight that the synthesis of nanohydroxyapatite (HA) from biogenic calcium carbonate sources such as mussel shells has garnered increasing attention for its sustainability and cost-effectiveness [1,2,3,4]. Using mussel shells lowers raw material costs and supports circular economy principles, but industrial translation requires control of process parameters, energy inputs, and reproducibility. Studies suggest feasibility, though costs related to calcination, purification, and post-processing remain critical [3,4]. In this study, HA particles were synthesized from *Perna perna* mussel shells via a wet chemical precipitation method followed by thermal treatment, yielding a material with nanoscale morphology, high crystallinity, and minor secondary phase content. Compared to Shavandi et al. (2014) [2], who utilized a rapid microwave-assisted method, the current approach required longer reaction times but achieved comparable phase purity and thermal stability, indicating the effectiveness of traditional synthesis routes when optimized. Moreover, Gupta et al. (2017) [20] employed HA in PLA-based bionanocomposites but did not detail secondary phases or purity, highlighting the added rigor of the present work in terms of material characterization. While Shariffuddin et al. (2013) [1] also derived HAP from mussel shells for photocatalytic applications, their product exhibited surface phosphate loss post-reaction, suggesting potential stability issues under certain conditions. Additionally, the presence of a controlled Ca/P ratio and neutral pH reflects an optimized synthesis process, which contrasts with literature reports of non-stoichiometric compositions in less refined protocols.

Overall, the current study not only confirms the feasibility of mussel shell-derived HA for biomedical use but also advances the field by integrating environmental valorization with thorough physicochemical and biological assessments, aligning with circular economy principles and clinical material standards. Also, the high crystallinity of HA aligns with its potential for bone regeneration, as crystalline hydroxyapatite closely mimics natural bone mineral [20,21]. The neutral to slightly alkaline pH of HA suspensions is favorable for biocompatibility, reducing the risk of inflammatory responses [22,25]. The presence of trace oxides in CaCO_3_, such as MgO and SiO_2_, may introduce impurities that affect its resorption kinetics in vivo.

Regarding the absence of antimicrobial effect of calcium carbonate, it can be inferred that the results observed with the analysis of antimicrobial activity here corroborate previous data in the literature. In fact, calcium carbonate has been used more as a carrier for antibiotics to produce an antimicrobial effect [41,42,43,44].

The MTT assay revealed that the mussel shell-derived CaCO_3_ exhibited excellent cytocompatibility with SHEDs, with all tested concentrations maintaining cell viability well above the cytotoxicity threshold defined by ISO 10993-5 [29] (70%). These findings suggest that the biogenic CaCO_3_ powder does not negatively affect mitochondrial activity or cell proliferation, even at elevated doses. The favorable response may be attributed to the bioactive nature of the calcium ions released and the absence of residual contaminants or toxic synthesis by-products.

The surface area of the synthesized HA (2.5–5.0 m^2^/g) is lower than that of some commercial HA powders (typically 5–60 m^2^/g). While the nanoscale morphology is beneficial for cellular interactions, the limited surface area could restrict protein adsorption and initial cell adhesion. Strategies such as adjusting precipitation parameters (e.g., precursor concentration, pH, and temperature), using surfactants to limit agglomeration, or employing templating approaches to generate hierarchical porosity have been shown to increase surface area and reactivity [4,16,20]. However, despite this lower value, our material still promoted protein adsorption and cellular mineralization, which indicates that surface reactivity was sufficient for biological interactions. These results highlight that surface chemistry and phase composition may play a more significant role than absolute surface area alone. The enhanced mineralization observed in SHEDs exposed to HA can be attributed to a synergistic mechanism involving (i) the presence of β-TCP, which releases Ca^2+^ and PO_4_^3−^ ions and promotes bioresorption, (ii) the surface chemistry of the particles, which supports cell adhesion, and (iii) the biphasic composition, which mimics natural bone remodeling processes. Together, these features likely explain the 2.4-fold increase in mineralization compared to controls. This supports the safe use of CaCO_3_ not only as a precursor for hydroxyapatite synthesis but also as a standalone biomaterial for applications in bone regeneration, dental materials, and bioactive fillers [4,8,16,18,20,25,45].

ARS results of the present study did show a positive effect of CaCO_3_ and HA on β-tricalcium phosphate release. These results implicate HA derived from mussel shells, depending on the synthesis route and the substrate used, may present traces of β-TCP in its structure [39,40]. This observation is supported by Rietveld-refined XRD data, which confirmed the presence of a biphasic HA/β-TCP system, with 13% of β-TCP phase detected in the synthesized material. Such consistency between ARS and structural analysis reinforces the influence of precursor choice and processing parameters on the final phase composition of the material [5,6,7,8,9,10,11,12,13,14,15,16,17,18,19,20,21,22,23,24,25,26,27,28,29,30,31,32,33,34,35,36,37,38,39,40,41,42,43,44,45,46,47].

In sum, the nanohydroxyapatite developed in this study, synthesized from calcium carbonate, shows great promise as a sustainable and cost-effective option for bone and dental tissue engineering. Despite the absence of antimicrobial activity, the mineralization tendency observed in SHEDs indicates its potential to support osteogenic and odontogenic processes, opening new possibilities for regenerative applications. To further enhance its biological performance, future investigations should explore optimization of synthesis parameters, particle size control, surface modifications, and doping with bioactive ions. A limitation of the present work is the lack of SEM–EDS mapping and DSC profiles obtained under identical temperature ranges due to equipment constraints. Although this prevented direct comparison, the thermal events observed remain consistent with the decomposition of CaCO_3_ and the stability of hydroxyapatite. Looking ahead, future studies will aim to repeat the DSC analyses under identical conditions, to evaluate long-term cytocompatibility, and to perform in vivo validation to confirm the osteogenic potential observed in vitro. Moreover, a comprehensive techno-economic assessment will be required to establish production costs, scalability, and industrial feasibility, ensuring the translation of this approach from laboratory research to practical biomedical applications.

## 5. Conclusions

We successfully synthesized nanohydroxyapatite from calcium carbonate, highlighting the potential for developing more sustainable biomaterials through the reuse of raw materials. Although the synthesized HA did not exhibit significant antimicrobial activity, it demonstrated biocompatibility as well as a tendency to promote mineralization in human exfoliated deciduous teeth stem cells (SHEDs). These results suggest a possible osteo/odontoinductive effect, although modest, indicating the need for further studies to better understand and enhance the biological performance of this promising material. Future applications may include its use as bone graft substitutes, dental composites, and drug delivery systems. Moreover, targeted modifications such as doping with trace elements or surface functionalization could further improve its biological performance and expand its clinical relevance.

## Figures and Tables

**Table 1:** Structural and microstructural parameters obtained from XRD-Rietveld Analyses of one of the batches.

Sample	Phases	Phase Fraction (%wt)	Lattice (Å)	Cry Size (nm)	Strain G
#1 CaCO_3_	Aragonite *Pmcn* (62)	96.12 (10)	a = 4.96575 (11)	76.7 (7)	0.0 (2)
			b = 7.96972 (17)		
			c = 5.74970 (10)		
	Calcite *R-3cH* (167)	3.88 (10)	a = 4.9893 (7)	60 (5)	0 (4)
			c = 17.056 (3)		
#1 HA	Degree of crystallinity (%)	75.28	-	-	-
	Hydroxyapatite *P63*/*m* (176)	86.3 (2)	a = 9.4283 (3)	83.0 (12)	0.152 (7)
			c = 6.8874 (3)		
	β-TCP *R-3cH* (161)	13.2 (2)	a = 10.4393 (6)	167 (16)	0.122 (16)
			c = 37.330 (3)		
	Brushite *Ia* (9)	0.52 (10)	a = 5.815 (15)	0 (110,000)	0.18 (11)

**Table 2:** Zeta potential (mV), particle size Z-Ave (d·nm), electrophoretic mobility (µm·cm/V·s), conductivity (mS/cm), and polydispersity index (PdI) measurements of CaCO_3_ and HA particles. Results are presented as individual values and mean ± standard deviation (SD).

Particles	Zeta Potential (mV)	Particle Size Z-Ave (d·nm)	Mobility (µm·cm/V·s)	Conductivity (mS/cm)	PdI
CaCO_3_	−18.00 ± 0.40	410.50 ± 17.04	−1.414 ± 0.031	0.05783 ± 0.00070	0.603 ± 0.150
HA	−18.47 ± 1.09	179.73 ± 20.24	−1.447 ± 0.084	0.02543 ± 0.00031	0.744 ± 0.074

**Table 3:** Physicochemical properties of synthesized HA.

Property	Unit	Value
Purity degree	%	~65–72
Crystallinity	%	~75–83
pH (10% solution)	–	7.0–8.0
Surface area	m^2^/g	2.5–5.0
Crystallite size	Nm	~60
Oxide residues (R_2_O_3_)	%	~7.16
HA 900 (TGA)	wt%	1004.62
Density	g/cm^3^	3.09–3.30

**Table 4:** Minimum inhibitory concentrations (MIC, mg/mL) determined for CaCO_3_ and HA particles against *E. faecalis*, *S. mutans*, *S. aureus*, and *C. albicans*.

Microorganisms	CaCO_3_	HA
*S. aureus*	>100 mg/mL	>100 mg/mL
*S. mutans*	>100 mg/mL	>100 mg/mL
*E. faecalis*	>100 mg/mL	>100 mg/mL
*C. albicans*	>100 mg/mL	>100 mg/mL

>100 mg/mL indicates that the minimum inhibitory concentration was higher than the maximum concentration tested.

## Data Availability

The original contributions presented in the study are included in the article, and further inquiries can be directed to the corresponding author.
